# Gate leakage current induced trapping in AlGaN/GaN Schottky-gate HFETs and MISHFETs

**DOI:** 10.1186/1556-276X-9-474

**Published:** 2014-09-08

**Authors:** Wen-Chia Liao, Yan-Lun Chen, Zheng-Xing Chen, Jen-Inn Chyi, Yue-Ming Hsin

**Affiliations:** 1 Department of Electrical Engineering, National Central University, No. 300, Jhongda Rd, Jhongli City, Taoyuan County 32001, Taiwan

**Keywords:** AlGaN/GaN heterostructure field-effect transistor (HFET), Dynamic on-resistance; Current collapse

## Abstract

This study examined the correlation between the off-state leakage current and dynamic on-resistance (R_ON_) transients in AlGaN/GaN heterostructure field-effect transistors (HFETs) with and without a gate insulator under various stress conditions. The R_ON_ transients in a Schottky-gate HFET (SGHFET) and metal-insulator-semiconductor HFET (MISHFET) were observed after applying various amounts of drain-source bias stress. The gate insulator in the MISHFET effectively reduced the electron injection from the gate, thereby mitigating the degradation in dynamic switching performance. However, at relaxation times exceeding 10 ms, additional detrapping occurred in both the SGHFET and MISHFET when the applied stress exceeded a critical voltage level, 50 V for the SGHFET and 60 V for MISHFET, resulting in resistive leakage current build-up and the formation of hot carriers. These high-energy carriers acted as ionized traps in the channel or buffer layers, which subsequently caused additional trapping and detrapping to occur in both HFETs during the dynamic switching test conducted.

## Background

Recently, AlGaN/GaN heterostructure field-effect transistors (HFETs) have been considered as a disruptive technology for high-power switching [1]. However, the degradation in dynamic switching performance is a crucial problem limiting the application of GaN-based HFETs [2,3]. To clarify the physical mechanisms, several studies have attributed this degradation in performance to two main sources. One source is the surface states associated with electrons injected from the gate. Injected electrons that are trapped in surface states form a negative potential that reduces the electrons in two-dimensional electron gas (2DEG) channels and acts as a ‘virtual gate’ in HFETs [4,5]. This degradation can be mitigated by using surface passivation techniques. The other source is the trapping of hot electrons in defective epitaxial layers, [6] which implies that the electrons in 2DEG channels can be driven by high electric field and trapped at barrier or buffer layers. Recent studies have indicated that a relationship exists between gate leakage-induced electron injection and defective epitaxial layers [7,8]. However, no study has clarified this leakage behavior and the involved trapping mechanism. Therefore, the behavior of dynamic on-resistance (R_ON_) transients in relation to V_DS_-dependent off-state leakage currents in HFETs under various stress conditions is discussed in this paper. Furthermore, the behavior of R_ON_ transients in HFETs with and without a gate insulator was compared, and the results revealed that a severe degradation in dynamic switching performance is due to a resistive leakage current formed by high electric field but not high electron injection.

## Methods

**Figure 1:**
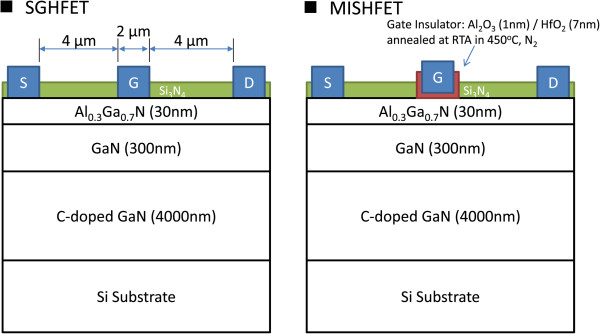
**Schematic cross-section and dimensions of the studied AlGaN/GaN HFETs.** The gate width is 50 μm.

Figure 1 shows the epitaxial layers and geometry of the Schottky-gate HFET (SGHFET) and metal-insulator-semiconductor HFET (MISHFET) examined in this study. The epitaxial layers and layout of these HFETs are identical. The layer structure comprises a 3.9-μm C-doped GaN buffer layer, 300-nm unintentionally doped (UID) GaN channel layer, 30-nm AlGaN barrier layer, and 1-nm UID GaN cap layer. The doping concentration of the C-doped buffer layer was 1 × 10^18^ cm^−3^. Both HFETs were fabricated based on the same layout and process flow, but different gate structures were used. The ohmic metal, Ti/Al/Ti/Au, was evaporated using an electron-beam evaporator, and it was annealed at 850°C for 30 s to form a low-contact resistance. The gate metal was a Ni/Au gate metal stack. The surface of these devices was passivated with a 200-nm silicon nitride layer, which was deposited using a plasma-enhanced chemical vapor deposition technique. The gate width, gate-source spacing, gate length, and gate-drain spacing were 50, 4, 2, and 4 μm, respectively. To obstruct the gate-injected electrons, an Al_2_O_3_/HfO_2_ (1 nm/6 nm) multistack gate insulator was deposited for the MISHFET at 250°C by using an atomic layer deposition technique (trimethylaluminum and water vapor were used as precursors). To enhance the quality of the gate insulator, postdeposition annealing was performed at 450°C for 1 min in an N_2_ ambient atmosphere.

## Results

**Figure 2:**
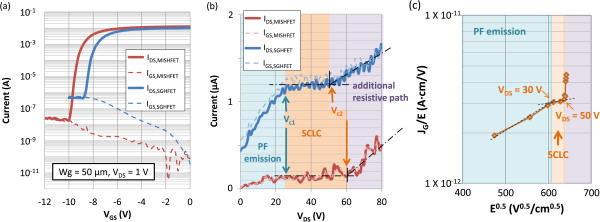
**Static characteristics of the studied devices. (a)** Transfer characteristics of the SGFET and MISFET. The gate leakage current in the MISFET was less than that in the SGHFET. **(b)** Off-state I-V curve of both HFETs. **(c)** Measured current density divided by the electric field versus the square root of the electric field for the SGHFET. The electric field was extracted from the simulation model.

The silicon substrate was floating during the HEMT characteristics tests in this study. The static transfer characteristics in Figure 2a show that the gate insulator effectively reduced the gate leakage current by more than one order in the pinch-off region. The drain current (I_DS_) on/off ratio of the SGHFET and MISHFET devices were 2.0 × 10^4^ and 3.3 × 10^5^, respectively. Figure 2b shows the off-state current-voltage (I-V) curves of both HFETs. The bias gate voltages of the SGHFET and MISHFET were −10 and −12 V, respectively. The leakage current in both HFETs was primarily from the gate. Two critical voltages, *V*_*c*1_ and *V*_*c*2_, can be determined from the characteristic curves. Under low electric field conditions, the leakage current increased in conjunction with the bias voltage. When applying voltages between *V*_*c*1_ and *V*_*c*2_, the leakage current cannot be influenced by increasing the V_DS_. Subsequently, the current increased when the bias voltage exceeded *V*_*c*2_. The *V*_*c*2_ values of the SGHFET and MISHFET were 50 and 60 V, respectively.

To explain the leakage mechanism, a technology computer-aided design simulation was performed using Atlas (Silvaco, Santa Clara, CA, USA) to examine the electric field. The epitaxial layers and layout of the simulation device were identical to those of the SGHFET (Figure 1). The carbon doping was modeled according to a compensation mechanism proposed by Armstrong et al. [9]. The *C*_*N*_-*C*_*Ga*_ states were autocompensated with *E*_*CGa*_ = 0.11 eV (donors) and *E*_*CN*_ = 3.28 eV (acceptors), and the concentrations of both *E*_*CGa*_ and *E*_*CN*_ were set at 1 × 10^18^ cm^−3^. Previously, Verzellesi et al. employed the C_DS_-V_DS_ measurement to verify the carbon-doping model used in this study [10].

Figure 2c shows a log-scale plot of *J*_*G*_/*E* as a function of *E*^0.5^ for the SGHFET. The electric field *E* was extracted from the near-surface electric field beneath the Schottky contact metal in the simulated device. Figure 2c shows that log(*J*_*G*_/*E*) is proportional to the square root of the electric field when the V_DS_ was less than 30 V. This result is in agreement with the Poole-Frenkel (PF) model, which has been widely studied in the Schottky-gate AlGaN/GaN HFETs [11,12]. The current associated with the PF effect is expressed as

(1) $$J_{G}=\mathrm{CE}\exp\left[-\frac{q\left(\emptyset_{t}-\sqrt{\mathrm{qE}/\pi \epsilon_{0}\epsilon_{s}}\right)}{k_{B}T}\right]\text{,}$$

where *E* denotes the electric field in the AlGaN barrier at the metal-semiconductor interface, ∅_*t*_ is the barrier height of the electron emission from the trapped state, *ϵ*_0_ represents the permittivity of free space, *ϵ*_*s*_ denotes the relative dielectric permittivity at high frequency, *T* is the temperature, *k*_*B*_ is Boltzmann’s constant, and *C* is a constant. From Equation 1, the current transport driven by the PF emission log(*J*_*G*_/*E*) is proportional to *E*^0.5^, as shown in Figure 2c; that is,

(2) $$\log\left(J_{G}/E\right)=\;\frac{q}{k_{B}T}\sqrt{\frac{\mathrm{qE}}{\pi \epsilon_{0}\epsilon_{s}}}-\frac{q\emptyset_{t}}{k_{B}T}+\log C$$

The PF effect implies that the injected carriers underwent a series of capture and emission processes. These processes prevent the applied electric field from effectively accelerating the injected carriers; consequently, an increased number of carriers are trapped near the surface. The high density of trapped carriers caused an electric field gradient to limit the current density. The current resulting from the presence of a space-charge effect is called space-charge-limited conduction (SCLC) [13]. However, when the applied V_DS_ exceeded *V*_*c*2_, the leakage current increased considerably, indicating that part of the carriers moved freely through the barrier layer. This characteristic curve was observed in both the SGHFET and MISHFET. However, the critical voltage *V*_*c*2_ of the SGHFET was approximately 50 V, as shown in Figure 2c, and a higher value of 60 V was observed in the MISHFET. These values are similar to those shown in Figure 2b.

In this study, the degradation in dynamic switching performance was determined by calculating the ratio of dynamic *R*_DS,on_ to *R*_DC_. The value of dynamic *R*_DS,on_ was obtained under test conditions in which V_DS,test_ and V_GS,test_ were respectively set to 1 and 0 V after applying the off-state stress. HFETs were stressed in high V_DS_ off-state (V_DS,stress_) for 1 s then synchronous switching V_GS_ and V_DS_ to the test condition by Agilent B1505 power device analyzer (Agilent Technologies, Santa Clara, CA, USA). The value of *R*_*DC*_ was obtained under test conditions in which V_DS_ and V_GS_ were respectively set at 1 and 0 V without applying the off-state stress. After each dynamic *R*_DS,on_ measurement, the initial condition of these devices can be fully recovered by shining microscope light for 10 min.

**Figure 3:**
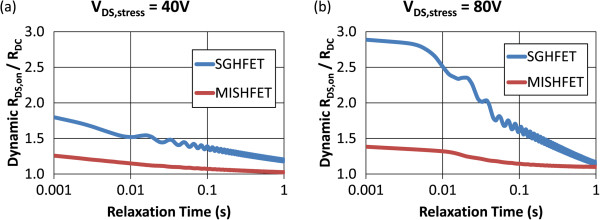
**R**_**ON **_**transient test results for HFETs under various off-state stress conditions. (a)** V_DS,stress_ = 40 V and **(b)** V_DS,stress_ = 80 V.

Figure 3 shows the test results of the R_ON_ transients in the HFETs under two stress conditions. When V_DS,stress_ was 40 V, the dynamic *R*_DS,on_/*R*_DC_ ratio of the SGHFET was higher than that of the MISHFET, although the recovery curves of both HFETs were similar. Furthermore, when V_DS,stress_ was 80 V, the dynamic *R*_DS,on_/*R*_DC_ ratio of the SGHFET decreased further from 2.5 to 1.5 when the relaxation time was between 10 and 100 ms. This strong recovery during this period caused a high dynamic *R*_DS,on_/*R*_DC_ ratio indicating that the dynamic switching performance of SGHFET was degraded substantially. The dynamic switching performance of the MISHFET was also degraded, although the decrease from 1.35 to 1.15 was comparatively less than that of the SGHFET. These results indicate that the gate insulator effectively mitigated the performance degradation; however, it did not suppress the additional trapping when high stress voltages were applied.

**Figure 4:**
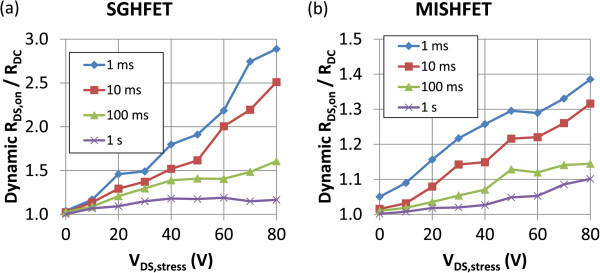
**Dynamic switching performance determined using various relaxation times after the HFETs underwent various stress conditions. (a)** SGHFET and **(b)** MISHFET.

Figure 4a,b depicts the V_DS,stress_ dependent recovery behavior of the SGHFET and MISHFET, respectively. The curves in these figures show the dynamic *R*_DS,on_/*R*_DC_ ratio at various relaxation times. The recovery behaviors can be separated into two groups based on the *V*_*c*2_ value; 50 V for the SGHFET and 60 V for MISHFET. The difference between these two groups was evident when the relaxation time was between 10 and 100 ms. As shown in Figure 4a, when the V_DS,stress_ of the SGHFET exceeded the value of *V*_*c*2_, the dynamic *R*_DS,on_/*R*_DC_ ratio decreased considerably when the relaxation time exceeded 10 ms, implying that a strong detrapping effect occurred at this time. Figure 4b shows that the MISHFET transients behaved similarly; moreover, the gate leakage current was also reduced, implying that the gate leakage-induced electron injection from the gate was not the primary cause of the detrapping process when the relaxation time exceeded 10 ms. The subsequent section explains how the detrapping process can be attributed to a high electric field.

## Discussion

**Figure 5:**
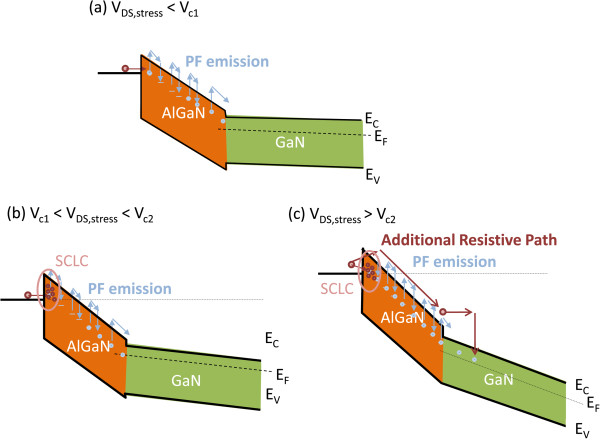
**Potential profiles corresponding to HFETs with V**_**DS,stress. **_**(a)** Below *V*_*c*1_, **(b)** between *V*_*c*1_ and *V*_*c*2_, and **(c)** greater than *V*_*c*2_.

This paper presents a model to explain the trapping mechanism based on the correlation between the off-state leakage current test results (Figure 2) and dynamic switching behavior (Figures 3 and 4). When the applied V_DS,stress_ was less than the critical voltage *V*_*c*1_, the injected carriers penetrated the AlGaN barrier layer through capture and emission processes, which can be explained by the PF effect (Figure 5a). When the applied V_DS,stress_ was between *V*_*c*1_ and *V*_*c*2_, the SCLC effect (Figure 5b) caused localized charges to occur at the surface of the epitaxial layers, thereby limiting the amount of injected carriers; consequently, the gate leakage current did not increase markedly when the V_DS_ was increased. However, devices under high V_DS,stress_ conditions can accumulate a considerable number of trapped carriers at surface states and/or in AlGaN barrier layer when the V_DS_ does not exceed *V*_*c*2_, which explains why the dynamic *R*_DS,on_/*R*_DC_ ratio continued to increase even when no excess carriers were injected into the channel. The gate insulator in the MISHFET effectively obstructed the electron injection from the gate, thereby mitigating the degradation in dynamic switching performance. Within this bias range, trapping behavior was primarily happening at the AlGaN barrier, which can be explained as ‘localized trapping’.

When the applied V_DS,stress_ exceeded the value of *V*_*c*2_ (Figure 5c), the amount of injected carriers was not limited by the localized trapping or SCLC effect. The root cause to overcoming this limitation can be attributed to either Fowler-Nordheim tunneling or deeper acceptor-like traps and emission mechanisms invoked by the PF effect. However, the characteristic curve in this V_DS,stress_ region was difficult to analyze because the electric field was not distributed in the AlGaN barrier layer alone; the depletion region in the 2DEG channel, which was extended under high V_DS_ conditions, should also be considered. Under high V_DS,stress_ conditions, the high electric field may have caused resistive leakage current, thereby causing part of the carriers to move freely through the AlGaN barrier layer. These free carriers can be driven by high electric fields that subsequently form hot carriers. These high-energy carriers could be trapped in the barrier, channel, or buffer layers; thus, a ‘global trapping’ effect occurred. Because the gate insulator mitigated the effect of the electric field on the barrier layer, the critical voltage of the MISHFET was higher than that of the SGHFET. However, the global trapping effect continued because high V_DS,stress_ applied to the MISHFET controlled the electron injection, which explains why a similar but less pronounced behavior was observed in the MISHFET (Figure 4b) as a result of the detrapping behavior.

## Conclusions

This study compared the off-state leakage current and characteristic curves of R_ON_ transients in AlGaN/GaN SGHFETs and MISHFETs to explain how the behavior of gate-injected electrons causes trapping and detrapping. The off-state leakage current follows PF effect for low-bias V_DS_. The gate insulator in the MISHFET effectively reduced the electron injection from the gate, thereby mitigating the degradation in dynamic switching performance. When the applied V_DS,stress_ exceeded the critical voltage, 50 V for the SGHFET and 60 V for MISHFET, resistive leakage current build-up caused part of the injected carriers to move freely through the barrier layer. These carriers can be accelerated by applying a high electric field to form hot carriers that act as ionized traps in the channel or buffer layers, thereby enhancing the trapping/detrapping effect in both SGHFETs and MISHFETs.

## Competing interests

The authors declare that they have no competing interests.

## Authors’ contributions

WCL designed and performed the experiments, analyzed the data, and drafted the manuscript. YLC and ZXC participated in the preparation of the devices. YMH and JIC supervised this study. All authors read and approved the manuscript.
